# Amygdala and fusiform gyrus temporal dynamics: Responses to negative facial expressions

**DOI:** 10.1186/1471-2202-9-44

**Published:** 2008-05-12

**Authors:** Jennifer C Britton, Lisa M Shin, Lisa Feldman Barrett, Scott L Rauch, Christopher I Wright

**Affiliations:** 1 Psychiatric Neuroimaging Research Program and Martinos Biomedical Imaging Center, Massachusetts General Hospital, Harvard Medical School, Boston, MA, USA; 2 Department of Psychology, Tufts University, Medford, MA, USA; 3 Department of Psychology, Boston College, Chestnut Hill, MA, USA; 4 Current: McLean Hospital, Belmont, MA, USA; 5 Division of Cognitive and Behavioral Neurology, Department of Neurology, Brigham and Women's Hospital, Boston, MA, USA

## Abstract

**Background:**

The amygdala habituates in response to repeated human facial expressions; however, it is unclear whether this brain region habituates to schematic faces (i.e., simple line drawings or caricatures of faces). Using an fMRI block design, 16 healthy participants passively viewed repeated presentations of schematic and human neutral and negative facial expressions. Percent signal changes within anatomic regions-of-interest (amygdala and fusiform gyrus) were calculated to examine the temporal dynamics of neural response and any response differences based on face type.

**Results:**

The amygdala and fusiform gyrus had a within-run "U" response pattern of activity to facial expression blocks. The initial block within each run elicited the greatest activation (relative to baseline) and the final block elicited greater activation than the preceding block. No significant differences between schematic and human faces were detected in the amygdala or fusiform gyrus.

**Conclusion:**

The "U" pattern of response in the amygdala and fusiform gyrus to facial expressions suggests an initial orienting, habituation, and activation recovery in these regions. Furthermore, this study is the first to directly compare brain responses to schematic and human facial expressions, and the similarity in brain responses suggest that schematic faces may be useful in studying amygdala activation.

## Background

Human faces provide key social and emotional information via the expressions portrayed. In a single encounter, an individual's facial expressions change rapidly, requiring a quick deduction of meaning. This ability to process facial expressions quickly or automatically is particularly advantageous when the expressions predict threat (e.g., fear or anger) [1]. Given the importance of processing social threat cues in facial expressions quickly, the meaning may be conveyed by several key features (e.g., raised eyebrows, angry eyes and gaping mouth).

Schematic faces, simple line drawings or caricatures of faces, extract these features from a complex facial expression. A schematic face capturing the key components of a facial expression may be useful in studies of emotion because the prototype is relatively devoid of confounding characteristics (e.g., ethnicity, age, attractiveness). Several studies have discovered that schematic faces still retain emotional meaning [2] and schematic faces activate brain structures involved in processing human facial expressions (e.g., amygdala, prefrontal cortex) [3], providing evidence that a simple representation of a facial expression can be used to study emotion.

It is well-established that the amygdala response habituates (i.e., decreases over time) to repeated presentations of human facial expressions; however, it is unclear whether the brain's response to schematic faces is maintained or habituates over time. Amygdala single cell recordings show reduced activity to repeated human faces [4]. Additionally, neuroimaging studies have reported early vs. late within-block habituation in the amygdala and hippocampal formation in response to repeated fearful and neutral human faces [5,6]. To our knowledge, only two studies have reported on habituation effects in response to schematic faces. In an event-related study involving both human and schematic faces, significant amygdala habituation was reported to schematic faces of anger relative to neutral in individuals with social phobia [7]. In a block-design study of healthy individuals, the amgydala response to schematic faces (angry, happy, and neutral) was maintained across time [3]; however, the presentation order (i.e., neutral blocks bracketing alternating emotional states) may have inhibited habituation.

In this fMRI block-design study, we examined the brain responses (i.e., amygdala and fusiform gyrus) to schematic and human facial expressions using a within-run facial expression (negative, neutral) and between-run face type (schematic, human) counterbalanced design. This design allowed examination of habituation and of face type in a single experiment without potentially confounding influences of presentation order. We hypothesized within-run habituation effects would be detected in response to the alternating blocks of schematic facial expressions as well as the human facial expressions in the amygdala.

## Results

### Face Recognition and Emotion Ratings

During the post-scanning recognition task, participants identified the faces viewed with high accuracy rates (human: 99.0% ± 4.2, schematic: 100% ± 0.0).

The negative faces [Schematic Negative: M = 3.4, SD = 1.3, Human Negative: M = 3.6, SD = 1.2] were rated as being more arousing than the neutral faces [Schematic Neutral: M = 1.9, SD = 1.3, Human Neutral: M = 1.9, SD = 1.3, expression effect: F(1,15) = 20.2, p < 0.004)]. No differences in arousal ratings between face type (i.e., schematic vs. human) were detected [face type effect: F(1,15) = 0.1, p > 0.77]. No interaction effects were noted [face type x expression: F(1,15) = 0.2, p > 0.66].

The negative faces [Schematic Negative: M = -2.6, SD = 1.3, Human Negative: M = -2.4, SD = 0.7] were rated as being more negative than the neutral faces [Schematic Neutral: M = 0.6, SD = 0.9, Human Neutral: M = -0.3, SD = 0.8, expression effect: F(1,15) = 254.0, p < 0.001]. The human faces tended to be rated more negatively than the schematic faces [face type effect: F(1,15) = 3.4, p = 0.09]. In addition, the valence rating difference between angry and neutral faces was greater for schematic faces than for human faces [face type x expression: F(1,15) = 11.8, p < 0.004]. This difference was due to greater negative valence ratings of the human neutral faces compared to more positive ratings of neutral schematic faces [t(15) = 3.1, p < 0.008].

### BOLD Activation

**Figure 1:**
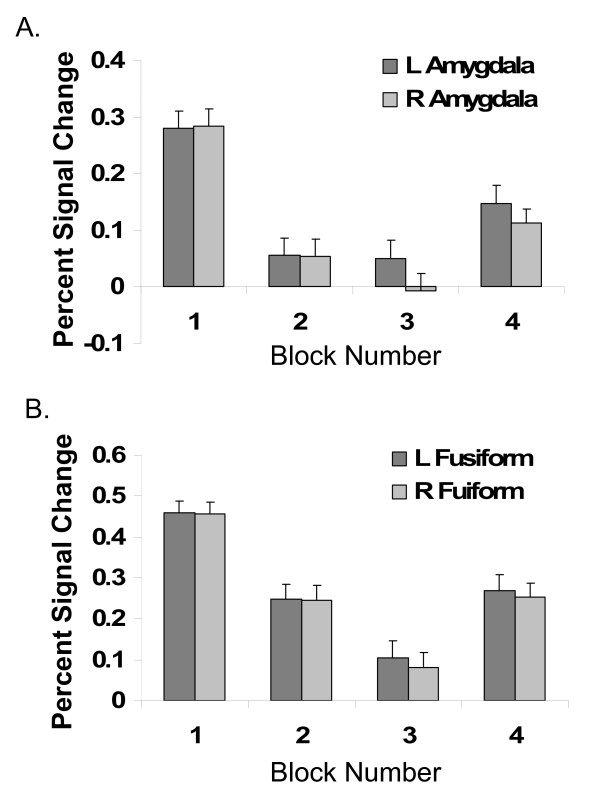
**Temporal dynamics of responses to facial expressions yield "U" pattern.** Percent signal change in brain activity in response to facial expressions compared to baseline across blocks in A) Amygdala and B) Fusiform Gyrus. L = Left, R = Right. Small bars indicate one standard error of the mean.

A temporal effect of responses across blocks within the run were detected in amygdala [time effect, Left: F(3,45) = 11.3, p < 0.001; Right: F(3,45) = 11.7, p < 0.001] and fusiform gyrus [time effect, Left: F(3,45) = 15.9, p < 0.001; Right: F(3,45) = 18.3, p < 0.001] (Figure 1). A significant quadratic response was detected in both regions [all F>27.6, p < 0.001].

Post-hoc tests were conducted using a significance threshold of p < 0.01 to correct for multiple comparisons and demonstrated a "U" pattern of activity. In the amygdala and fusiform, the response in the initial block (block 1) was greater than the other blocks (blocks 2, 3, and 4) [all regions: T>2.8, p < 0.01]. Contrary to the amygdala [both hemispheres: T<1.2, p > 0.2], the responses in the fusiform gyrus tend to progressively decline (block 2> block3) [both hemispheres: T>2.4, p < 0.03]. In addition, a trend towards significant region (amygdala, fusiform) x time (block 2, block 3) interaction was detected in the left [F(1,15)>5.1, p < 0.04] and the right hemispheres [F(1,15)>3.8, p < 0.07]. Responses to block 4 were greater than block 3 significantly in the right amygdala and bilateral fusiform gyrus [all regions: T>2.9, p < 0.01] and at trend-level significance in the left amygdala [T>2.2, p < 0.04].

**Figure 2:**
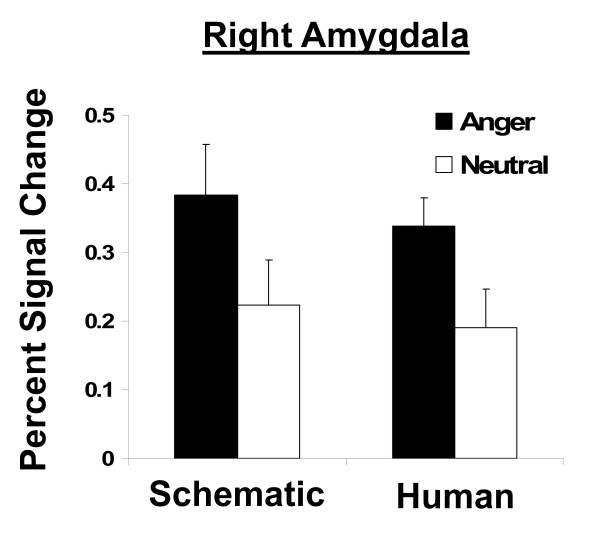
**Right Amygdala response to emotion in initial block of facial expressions.** Percent signal change in amygdala activity in response to facial expressions compared to baseline for first block of each run. Greater responses were elicited to negative faces compared to neutral faces in the right amygdala [F(1,15)>7.8, p < 0.01]. No significant main effect of face type (schematic, human) was noted.

In the right amygdala, a trend emotion x time effect was significant [F(3, 45) = 0.06]. Because the initial block yielded the largest response and was subsequently followed by a habituated response, the responses in the first time block were investigated further for valence effects. Greater responses were elicited to negative faces compared to neutral faces [F(1,15)>7.8, p < 0.01]. (Figure 2).

There were no other significant effects (i.e., face type) in the amygdala. No significant valence, face-type main effets or interactions were found in the fusiform gyrus. [all effects: F<1.7, all p > 0.2].

## Discussion

Within each run, the amygdala and the fusiform gyrus showed a "U" response pattern with the initial and final blocks eliciting the greatest activation to a repeated facial expression. The amygdala profile may reflect an initial orienting response, then habituation, followed by recovery of activation in the final block. A similar "U" pattern was observed in skin conductance response (SCR) and late-phase SCR-associated left amygdala response to repeated fearful faces [8]. Like fear, anger is highly arousing and may prompt a similar orienting response and skin conductance response. The activation recovery may be due to emotional priming [8]. Alternatively, it may reflect spontaneous recovery or reinstatement. Vigilance maintenance via a system reset, even in the absence of imminent threat, may be an important survival function [9]. Consistent with this notion, primate electrophysiological data demonstrate that neuronal populations within the amygdala respond maximally to novelty, show decreased activation with familiarity (i.e., habituation), and reset (i.e., show activation again) after limited number of repeated stimulus presentations [4].

In this study, fusiform gyrus activation followed this "U" pattern in response as well; however, a trend towards different temporal patterns are observed in the amygdala and fusiform gyrus. In the amygdala, habituation occurs rapidly; whereas, in the fusiform gyrus, habituation occurs more gradually. This delayed recovery may be explained by enhanced modulation of the amygdala or a slower resetting system of the fusiform gyrus.

Negative faces are discriminated from neutral faces in the right amygdala. Our findings replicate previous work showing that the right amygdala, responds to angry relative to neutral faces [10,11]. In this study, the differential amygdala response to facial expressions was present only during the early time period, suggesting that it is related to the amygdala orienting response. Although some studies report fusiform gyrus activation to emotional faces relative to neutral faces, we did not detect such an effect. It may be that differential fusiform gyrus activation to emotional (vs. neutral) faces is task-dependent. The fusiform gyrus responds more non-selectively to facial stimuli in the context of limited-attentional demands (e.g., passive viewing of repeated facial expressions) [12], yet exhibits a selective or differential pattern of activation when increased attention to face emotional content is required [13]. In fact, the existence of projections from the amygdala to the fusiform cortex suggest that the amygdala may modulate the sensory processing stream according to the salience of the target visual stimulus [14].

Interestingly, no significant differences between schematic and human faces were detected in the amygdala and fusiform gyrus. In a recent study, the amygdala response to human and avatar (or computer-generated faces) was similar, yet the fusiform showed a greater response to human faces [15]. For studying the amygdala, it appears that there is some utility to this response similarity between human and face representations (e.g. schematic or avatar faces). Schematic and avatar faces may be useful to study emotion perception because the key facial features that underlie the neural activation are relatively isolated from stimulus features like race/ethnicity and gender, which may increase the variability in responses. It is also important to note that schematic and avatar faces may be useful in answering different questions concerning emotion that take advantage of the static or moveable representations (e.g. brain responses to key facial features and brain responses to social emotional interactions, respectively).

This study has some potential limitations. Evaluating the temporal dynamics of neural responses is dependent on the time scale examined. In this study, within-run habituation effects were investigated; however, other time scales (e.g., between-run, within-block) may show different effects. Only angry faces were used to represent negative faces. Future studies should examine the temporal dynamics of other expressions (e.g., fearful, sad), including positive expressions (e.g. happy). Our findings suggest schematic and human faces elicit generally similar responses in the amygdala and fusiform gyrus; however, replication in a larger sample is needed. Schematic faces reduce expressions to line drawings and a single exemplar was used in this study. While using a single exemplar may be problematic, it does diminish confounds due to variability in human facial expressions. Finally, although using ROI-based analysis is a more powerful approach for detecting differences in specific a priori regions (i.e., amygdala and fusiform gyrus), this approach does not allow the observation of other regions that may also respond to these stimuli.

## Conclusion

In summary, it appears that both the amygdala and fusiform gyrus responses to facial expressions do habituate over time; however, the "U" pattern suggests that the responsivity of these structures resets, possibly to allow attentional reengagement with repeatedly presented stimuli. Future studies with larger samples should investigate whether this pattern discriminates between emotions or stimulus type.

## Methods

### Participants

Sixteen participants were studied (8 females, 8 males; M = 26.7, SD = 4.7, Range = 22–41 years of age). All participants were Caucasian and right-handed determined by the Edinburgh Handedness Inventory [16], free of psychoactive medications and medical, neurological or psychiatric illness. Beck Depression Inventory (BDI) [17] and Beck Anxiety Inventory (BAI) [18] scores were in the normal range (BDI: M = 0.9, SD = 1.8, BAI: M = 2.4, SD = 2.2).

This study was approved and conducted in accordance with guidelines established by the Partners Human Research Committee. Written informed consent was obtained from each participant.

### Stimuli

**Figure 3:**
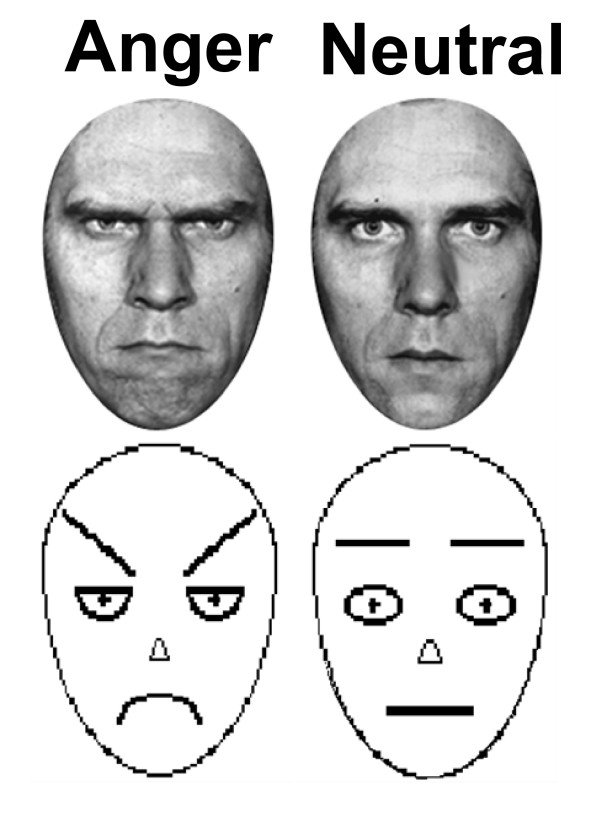
Human and Schematic Faces.

Participants viewed human faces [19] and schematic faces [20] (Figure 3). To match the perceptual stimulus features, the human and schematic faces were presented in black and white and scaled so the face silhouette (excluding hair) was identical between stimuli. The face stimuli were displayed using standardized software (MacStim 2.5.9) and a Sharp XG-2000 V color LCD projector (Osaka, Japan).

Four different human face identities (two male and two female), each displaying both negative (i.e. angry) and neutral expressions, and one neutral and one negative (i.e. angry) schematic face were used throughout the experiment. Since the schematic faces only have a single identity, each participant viewed human faces of a single identity. As the schematic faces do not have an intrinsic gender, the gender of the human faces viewed was counterbalanced across participants.

### Procedure

All participants viewed four 5-minute 24-second runs of faces. Two consecutive runs of schematic faces and two consecutive runs of human faces were counterbalanced across participants. Within each run, four blocks of negative and four blocks of neutral were counterbalanced within and across participants. Each 24-second face block was separated by a 12-second low-level fixation. All runs were bracketed by a 24-second low-level fixation. This design improved upon earlier studies and allowed for better assessment of habituation, given that: 1) neutral blocks were included throughout the run, 2) fixation blocks were interleaved and 3) the design was fully counterbalanced. Within the face blocks, participants viewed a single human or schematic face, repeatedly shown for 200 ms with a 300 ms interstimulus interval.

Before each run, participants were instructed to remain awake and alert and look at the faces at eye level. Immediately after scanning, each participant was given a face recognition form, displaying target faces as well as eight distracters: two schematic (happy and scheming expressions) and six human (three different identities, each displaying neutral and negative expressions). Additionally, participants rated the schematic and human faces according to arousal value (low-high: 0–6) and valence (negative-positive: -3 to +3).

Repeated measures analysis of variance (ANOVA) was used to assess for significant differences in subjective ratings. Significant main effects and interaction effects were determined using a p < 0.05 threshold.

### Image Acquisition

A Sonata 1.5 Tesla whole-body high-speed imaging device equipped for echo planar imaging (EPI) (Siemens Medical Systems, Iselin NJ) was used with a 3-axis gradient head coil. After acquiring an automated scout image and optimizing field homogeneity via localized shimming procedures, a high resolution 3D MPRAGE sequence (TR/TE/flip angle = 7.25 ms/3 ms/7°, 1.3 mm in-plane resolution, and 1 mm slice thickness) was collected for anatomical registration and normalization. Then, T1-EPI (TR/TE/flip angle = 8 sec/39 ms/90°) and T2-weighted (TR/TE/flip angle = 10 sec/48 ms/120°) sequences were gathered to monitor scanner function and assist with anatomical registration. Functional MRI images (i.e., blood-oxygenation-level-dependent or BOLD images) were acquired using a gradient echo T2*-weighted sequence (TR/TE/flip angle = 2.4 sec/40 ms/90°), discarding the first four acquisitions to allow longitudinal magnetization to reach equilibrium. Twenty-four coronal slices (slice thickness: 7 mm, 1 mm skip, voxel size: 3.125 × 3.125 × 8 mm) were acquired perpendicular to the ac-pc line. The acquisition parameters were used to minimize susceptibility in medial temporal lobe regions [21].

### fMRI Data Analyses

Functional MRI data were analyzed using the standard processing stream of the Martinos Center for Biomedical Imaging [22]. The functional runs were motion corrected using an AFNI-based algorithm [23,24]. The average motion vector across all runs after correction was <1 mm and showed no significant difference between schematic and human face runs. The functional data were spatially smoothed (full-width-half-maximum = 7 mm) using a 3D Gaussian filter and intensity normalized to correct for global signal changes. Data processing included 1) 2^nd^-order polynomial drift correction to account for low-frequency drift and 2) removal of temporal autocorrelation by whitening [25]. The functional images were aligned to the 3D structural image. During registration, the raw functional data from each participant were visualized in anatomical space to determine that the amygdala BOLD signal was not obscured by susceptibility artifact. No subjects were excluded on this basis.

Functional images were averaged across participants according to expression (neutral, negative). For each expression, averages were made for each block (1,2,3,4) to assess temporal aspects. The averages were collapsed across the runs for each face type (schematic, human) separately. Using an anatomically defined region of interest (ROI)-based approach, each participant's left and right amygdala were manually traced on the participant's high resolution 3D MPRAGE sequence by a trained technician of the Massachusetts General Hospital's Center for Morphometric Analysis [26]. The fusiform gyrus was defined using similar methods and used as a comparison region. The anatomical tracings were used to extract functional data from each participant's selectively averaged BOLD images. The percent signal change for each condition versus fixation was calculated for each participant, and this information was entered into repeated measures ANOVA with within-subject factors: face type (human, schematic), expression (neutral, negative), time (block 1, block 2, block 3, block 4). A separate repeated measures ANOVA was used for each anatomic ROI. Main effects and interaction effects were examined. Significance was determined using p < 0.05. Where appropriate, the sources of significant findings were evaluated using post-hoc tests and multiple-comparison correction (i.e. using a reduced p-value threshold).

## Authors' contributions

JCB conducted the analysis and drafted the manuscript. LMS, LFB, SLR helped design the study and revise the manuscript. CIW conceived of the study, coordinated its completion, and helped to draft the manuscript.

## References

[B1] Mather M, Knight MR (2006). Angry faces get noticed quickly: threat detection is not impaired among older adults. J Gerontol B Psychol Sci Soc Sci.

[B2] Aronoff J, Barclay AM, Stevenson LA (1988). The recognition of threatening facial stimuli. J Pers Soc Psychol.

[B3] Wright CI, Martis B, Shin LM, Fischer H, Rauch SL (2002). Enhanced amygdala responses to emotional versus neutral schematic facial expressions. Neuroreport.

[B4] Wilson FA, Rolls ET (1993). The effects of stimulus novelty and familiarity on neuronal activity in the amygdala of monkeys performing recognition memory tasks. Exp Brain Res.

[B5] Wright CI, Fischer H, Whalen PJ, McInerney SC, Shin LM, Rauch SL (2001). Differential prefrontal cortex and amygdala habituation to repeatedly presented emotional stimuli. Neuroreport.

[B6] Wedig MM, Rauch SL, Albert MS, Wright CI (2005). Differential amygdala habituation to neutral faces in young and elderly adults. Neurosci Lett.

[B7] Straube T, Kolassa IT, Glauer M, Mentzel HJ, Miltner WH (2004). Effect of task conditions on brain responses to threatening faces in social phobics: an event-related functional magnetic resonance imaging study. Biol Psychiatry.

[B8] Williams LM, Brown KJ, Das P, Boucsein W, Sokolov EN, Brammer MJ, Olivieri G, Peduto A, Gordon E (2004). The dynamics of cortico-amygdala and autonomic activity over the experimental time course of fear perception. Cogn Brain Res.

[B9] Kesler-West ML, Andersen AH, Smith CD, Avison MJ, Davis CE, Kryscio RJ, Blonder LX (2001). Neural substrates of facial emotion processing using fMRI. Brain Res Cogn Brain Res.

[B10] Fischer H, Sandblom J, Gavazzeni J, Fransson P, Wright CI, Backman L (2005). Age-differential patterns of brain activation during perception of angry faces. Neurosci Lett.

[B11] Whalen PJ, Shin LM, McInerney SC, Fischer H, Wright CI, Rauch SL (2001). A functional MRI study of human amygdala responses to facial expressions of fear versus anger. Emotion.

[B12] Gauthier I, Tarr MJ, Moylan J, Skudlarski P, Gore JC, Anderson AW (2000). The fusiform "face area" is part of a network that processes faces at the individual level. J Cogn Neurosci.

[B13] Breiter HC, Etcoff NL, Whalen PJ, Kennedy WA, Rauch SL, Buckner RL, Strauss MM, Hyman SE, Rosen BR (1996). Response and habituation of the human amygdala during visual processing of facial expression. Neuron.

[B14] Amaral DG, Behniea H, Kelly JL (2003). Topographic organization of projections from the amygdala to the visual cortex in the macaque monkey. Neuroscience.

[B15] Moser E, Derntl B, Robinson S, Fink B, Gur RC, Grammer K (2007). Amygdala activation at 3T in response to human and avatar facial expressions of emotions. J Neurosci Methods.

[B16] Oldfield RC (1971). The assessment and analysis of handedness: The Edinburgh Inventory. Neuropsychologia.

[B17] Beck AT, Steer RA, Corporation TP (1990). Beck Anxiety Inventory Manual.

[B18] Beck AT, Ward CH, Mendelson M, Mock J, Erbaugh J (1961). An inventory for measuring depression. Arch Gen Psychiatry.

[B19] Ekman P, Friesen WV (1976). Pictures of Facial Affect.

[B20] Ohman A, Lundqvist D, Esteves F (2001). The face in the crowd revisited: a threat advantage with schematic stimuli. J Pers Soc Psychol.

[B21] Wright CI, Martis B, Schwartz CE, Shin LM, Fischer HH, McMullin K, Rauch SL (2003). Novelty responses and differential effects of order in the amygdala, substantia innominata, and inferior temporal cortex. Neuroimage.

[B22] Freesurfer software. http://surfer.nmr.mgh.harvard.edu.

[B23] AFNI software. http://afni.nimh.nih.gov/.

[B24] Cox RW (1996). AFNI: software for analysis and visualization of functional magnetic resonance neuroimages. Comput Biomed Res.

[B25] Burock MA, Dale AM (2000). Estimation and detection of event-related fMRI signals with temporally correlated noise: a statistically efficient and unbiased approach. Hum Brain Mapp.

[B26] Caviness VS, Kennedy DN, Richelme C, Rademacher J, Filipek PA (1996). The human brain age 7-11 years: a volumetric analysis based on magnetic resonance images. Cereb Cortex.

